# Preparation of Calcium-Chelating Peptides from Squid Skin and Evaluation of Calcium Absorption Capacity in Caco-2 Cell Monolayer Model

**DOI:** 10.3390/foods14091594

**Published:** 2025-04-30

**Authors:** Jihao Zeng, Xue Bai, Yongli Zhang, Qianyu Le, Jinhong Wu, Huiyun Chen

**Affiliations:** 1 Institute of Agricultural Product Processing Research, Ningbo Academy of Agricultural Science, NO.19 Dehou Street, Ningbo 315040, China; melozeng0127@outlook.com; 2 Department of Food Science and Engineering, School of Agriculture and Biology, Shanghai Jiao Tong University, Shanghai 200240, China; nndbaixue@sina.com (X.B.); yongliz@163.com (Y.Z.); qianyu.le@outlook.com (Q.L.)

**Keywords:** squid skin, calcium peptide chelation, oxidation resistance, calcium absorptivity

## Abstract

To develop a highly bioavailable calcium supplement, this study utilized Peruvian squid (*Dosidicus gigas*) skin as a raw material. Through alkaline protease hydrolysis and enzymatic membrane reactor separation, three molecular weight fractions of squid skin peptides were obtained, followed by calcium ion chelation to synthesize calcium-chelating peptides (CCPs-SS). Systematic characterization revealed that the less than 1 kDa fraction of CCPs-SS exhibited superior antioxidant capacity (82.18%) and calcium chelation efficiency (77.14%) in cellular models compared to higher molecular weight counterparts. Optimal synthesis conditions were identified as 60 °C, pH 9, and 12 mg/mL calcium chloride concentration. Post-chelation analyses demonstrated significant physicochemical alterations for CCPs-SS: ζ-potential shifted from −18.4 mV to −10.47 mV, while particle size increased from 476.75 nm to 664.4 nm. Notably, membrane separation enhanced phenylalanine and leucine molar concentrations by 25.5% and 57.6%, respectively, suggesting structural modifications that potentiate bioactivity. These findings demonstrate an innovative strategy for converting squid processing byproducts into functional nutraceuticals, which not only addresses calcium deficiency challenges but also promotes resource sustainability by utilizing waste materials.

## 1. Introduction

Squid is a high-protein, low-fat nutritional seafood. With the rapid development of the squid processing industry in China, the recycling and reuse of squid processing by-products, such as fish skin, viscera and cartilage, have become the focus of researchers. Squid skin accounts for 8% to 13% of the total waste and is very rich in resources. As the protein with the highest content in squid skin, the small molecule peptide obtained by further hydrolysis of collagen is not only beneficial to human body absorption, but also has a variety of physiological activities such as antioxidant activity, ACE inhibition activity, tumor cell inhibition activity and anti-aging [1,2]. Extracting collagen peptides from discarded squid skin and applying them to a functional food system can not only relieve environmental pressure, and increase the added value of squid by-products, but also broaden the source of foodborne bioactive peptides [3]. However, the utilization of squid skin collagen peptides still faces many problems, such as fishy taste, dark color, poor stability and low bioavailability, which limit their application in the food industry.

In addition to biological activity, squid skin peptides can also chelate with metal ions, and the chelating activity is good. Calcium is the most abundant inorganic element in the human body [4], accounting for 1.5–2.2% of total body weight [5,6]. Calcium also plays an important role in intracellular metabolism, bone growth, blood clotting, nerve conduction, muscle contraction, and cardiac function [7]. Calcium deficiency may result in osteoporosis, rickets, and even osteomalacia. Ca^2+^ has served as the main source of calcium supplements for humans in recent years, thereby limiting the bioavailability of calcium from dietary supplements. Calcium-binding peptides can form complexes with calcium to improve its absorption and bioavailability. Amino acid chelating calcium has been studied often. The groups that can bind calcium ions mainly include the amino acid α-amino group, side chain ligand group and carboxyl group. In addition, carbonyl groups or deprotonated amino nitrogen on peptide bonds can also coordinate with calcium ions to form a more stable structure [8].

Peptide calcium chelates are usually centered on calcium ions, and other atoms bind to amino acids and form a five or six-membered ring structure with calcium ions, which is relatively stable [9,10]. The body absorbs amino acids or small peptides after chelating with calcium ions. Peptide-calcium chelates can be absorbed in the small intestine in the form of small molecule peptides through the peptide absorption pathway [11], including transcellular pathway transport peptides [12,13] and paracellular pathway transport [14,15]. The human body can not only absorb calcium in chelated form, but also release calcium ions effectively, and peptide calcium chelates can be used as a new type of calcium supplement.

Current research on squid-derived collagen peptides and their metal chelates remains limited. A critical knowledge gap lies in understanding their bioavailability and physiological efficacy, which directly impacts their potential applications in functional foods and nutraceuticals. To address this, we systematically investigate squid skin byproducts through three key dimensions: (1) bioactive properties, (2) optimized preparation protocols, and (3) cellular absorption kinetics. This multidisciplinary approach establishes a mechanistic framework to advance the utilization of collagen peptide chelates in health-promoting food systems.

## 2. Materials and Methods

### 2.1. Materials

The squid skin used in this study was a processing by-product of Peruvian squid. It was caught in the East China Sea by Ningbo Fei Run Marine Biological Polytron Technlolgies Inc. After being sun-dried and dehydrated, it was stored for future use and kept in a cool place.

#### Preparation of Collagen Peptides with Different Molecular Weights from the Squid Skin Hydrolysate

A mixed solution was prepared by dissolving 6 g of sodium chloride and 1.8 g of acetone (Aladdin Shanghai, China) in 1080 mL of water. Squid skin (120 g) was soaked in this solution for 12 h. The soaked squid skin was then transferred to a 2% bromelain solution and hydrolyzed in a hydrolysis reactor at 55 °C for 120 min. After hydrolysis, the enzyme was inactivated by heating the hydrolysate at 100 °C for 3 min.

The hydrolysate was subsequently processed in an enzymatic membrane reactor (GS-05EMR, Suzhou, China). The membrane separation device was operated at a flow rate of 10 L/h and a membrane pressure of 0.6 bar. The hydrolysate obtained was then ultra-filtrated by three kinds of ultrafiltration membranes (Polysulfone ultrafiltration flat film), including 1, 3 and 5 kDa. Then, three peptide components, including 1 K (MW < 1 kDa), 3 K (1–3 kDa) and 5 K (3–5 kDa), were collected, concentrated, and freeze-dried.

### 2.2. Characterization of Collagen Peptides with Varied Molecular Weights

#### 2.2.1. Amino Acid Composition Analysis

The amino acid composition of calcium-chelating peptides was determined using acid hydrolysis followed by high-performance liquid chromatography (HPLC). Briefly, peptide samples (2 mg) were hydrolyzed with 6 M HCl (containing 0.1% phenol to minimize oxidative degradation) at 110 °C for 24 h under vacuum-sealed conditions. After hydrolysis, the residual HCl (Aladdin Shanghai, China) was removed by evaporation under nitrogen flow, and the hydrolysate was reconstituted in 0.02 M HCl.

The amino acid derivatives were separated using a reversed-phase C18 column (Agilent ZORBAX Eclipse Plus, 4.6 × 250 mm, 5 μm) with a gradient elution system consisting of solvent A (0.1% trifluoroacetic acid in water, Sigma-Aldrich Corp, GR) and solvent B (0.1% trifluoroacetic acid in acetonitrile, Sigma-Aldrich Corp, GR). Post-column derivatization with ninhydrin or o-phthalaldehyde (OPA) was employed for fluorescence detection (excitation 340 nm, emission 455 nm). Quantification was achieved by comparing peak areas against a standard amino acid mixture (Sigma-Aldrich AAS18, Saint Louis, MO, USA), and cysteine/methionine contents were verified by performic acid oxidation prior to hydrolysis.

#### 2.2.2. Hydroxyl Radical Scavenging Activity Assay

The extraction process was conducted with a liquid-to-solid ratio of 1:10 (*v*/*w*). Precisely 0.1 g of freeze-dried powder was weighed and homogenized in an ice bath with 1 mL of extraction solvent. The homogenate was then centrifuged at 10,000 g for 10 min at 4 °C, and the supernatant was collected and stored on ice for subsequent analysis. For the preparation of the enzymolysis solution, the stock solution was diluted to achieve concentrations ranging from 10 to 50 mg/mL. The hydroxyl radical scavenging activity of the enzymolysis solution derived from squid skin was assessed across these concentrations. The hydroxyl radical scavenging activity was measured using a hydroxyl radical scavenging assay kit (Leagene, Beijing, China).

#### 2.2.3. Determination of Ca-Chelating Rate

Lyophilized squid skin peptides with pre-fractionated molecular weights (1 g) were dissolved in 5 mL of deionized water. A 4 mL aliquot of the peptide solution was mixed with 1 mL of CaCl_2_ (Aladdin Shanghai, China) solution (12 mg/mL) in a centrifuge tube, yielding a final reaction volume of 5 mL. The pH was adjusted to 9.0 using 0.1 M NaOH/HCl (Aladdin Shanghai, China), and the mixture was incubated in a water bath at 70 °C for 60 min under constant agitation (200 rpm). After cooling to room temperature (25 °C), calcium-chelated peptides were precipitated by adding nine volumes of ice-cold absolute ethanol (*v*/*v*). The solution was centrifuged at 6000× *g* for 10 min at 4 °C. The supernatant was discarded, and the pellet was collected, frozen at −80 °C for 2 h, and lyophilized (Christ Alpha 1-4 LDplus) to obtain the final calcium-chelated peptide powder (CCPs-SS). Calcium content was quantified via atomic absorption spectrophotometry, while protein content was determined using the Kjeldahl method. Calcium chelating capacity was calculated as the mass ratio of calcium to squid protein.

$$B\left(\%\right)=\frac{m_{calcium}}{m_{protein}}\times 100$$

where m_calcium_ represents the mass of calcium in the sample and m_protein_ represents the total mass of squid peptides involved in the reaction.

### 2.3. Preparation and Optimization of CCPs-SS

A 0.5% (*w*/*v*) squid collagen peptide solution was prepared and mixed with an equal volume of 0.5% (*w*/*v*) calcium chloride solution under the reaction conditions outlined in Table 1 and Table 2, with calcium chelation capacity as the evaluation metric. Following the reaction, absolute ethanol (9× reaction volume) was added to the mixture and incubated for 30 min. The resulting precipitate was isolated by centrifugation (10,000× *g*, 4 °C, 10 min), collected, and lyophilized to obtain the chelating calcium peptide of squid skin (CCPs-SS) [12].

### 2.4. Structural Characterization of the CCPs-SS

#### 2.4.1. Scanning Electron Microscopy (SEM) Analysis

According to the method of Jiao Yan et al. [16], the CCPs-SS sample was dissolved in water, and then an appropriate amount of the sample was dropped onto the copper mesh by a dropper and treated with plasma. After the water was dried, the microstructure of the chelated peptide was observed by transmission electron microscope (Rise-Magna, Tescan, Brno, Czech Republic).

#### 2.4.2. Particle Size and Potential Analysis

The CCPs-SS samples were analyzed using a Nano-90 dynamic light scattering (DLS) analyzer (Malvern Panalytical, UK) by Yuan et al. [17]. Triplicate measurements of hydrodynamic diameter, polydispersity index (PDI), and zeta potential were conducted at 25 °C with a detection angle of 90°.

#### 2.4.3. Fourier Transform Infrared (FTIR) Spectroscopy

Freeze-dried peptide powders were subjected to FTIR analysis (Nicolet 6700, Thermo Fisher Scientific, Waltham, MA, USA) using the method of Liao and Zhang [18,19]. Spectra were acquired over 500–2000 cm^−1^ with 200 scans at 4 cm^−1^ resolution. Baseline correction and peak deconvolution were performed using OMNIC 9.2 software (Thermo Fisher Scientific, Waltham, MA, USA).

### 2.5. Assessment of the Calcium Absorption Capacity of CCPs-SS Using Caco-2 Cell Monolayers Model

#### 2.5.1. Cultivation of Caco-2 Cells

Cells were seeded in 25 mL culture flasks (12.5 cm^2^) and incubated at 37 °C with 5% CO_2_ [20]. The medium was changed every other day. The complete medium comprised 88% MEM (Gibco, Carlsbad, CA, USA), 1% nonessential amino acids, 1% antibiotics, and 10% fetal bovine serum. Cell growth was monitored using an inverted microscope. When the cells reached 80–90% confluence, the medium was removed, and the cells were washed twice with PBS. After blotting out the PBS, 1 mL of 0.25% trypsin was added, and the cells were incubated for 3 min [21]. The cells were then detached from the flask walls, resuspended, and transferred to a 10 mL centrifuge tube. Centrifugation was performed at 800–900 rpm for 3 min [22,23]. The supernatant was discarded, and the cell pellet was resuspended in an appropriate volume of complete medium, taking care to avoid bubble formation. The cells were then transferred to a new culture bottle for further growth and prepared for subsequent experiments after two passages.

#### 2.5.2. Establishment of Caco-2 Cell Model

Caco-2 cells in the logarithmic growth phase were seeded onto polyester membranes of 6-well Transwell^®^ inserts at a density of 3 × 10^5^ cells/mL (1.5 mL per insert). Prior to cell seeding, the inserts and basolateral chambers were pre-equilibrated by adding 3.5 mL of complete medium to the lower compartment and 1 mL to the apical chamber, followed by incubation at 37 °C under 5% CO_2_ for 2 h to enhance membrane hydration and cell adhesion [24,25,26]. The culture medium was replaced with a fresh complete medium every 48 h throughout the differentiation period.

#### 2.5.3. Cytotoxicity Assay

Caco-2 cells in the logarithmic growth phase were trypsinized, resuspended in a complete medium, and counted using a hemocytometer under an optical microscope. The cell density was adjusted to 3 × 10^4^ cells/mL, and 100 μL of the suspension was seeded into each well of a 96-well plate. After 12 h of incubation (37 °C, 5% CO_2_), the medium was aspirated, and replaced with 100 μL of test solutions (prepared in MEM medium) at concentrations of 0.01, 0.02, 0.1, 0.2, 0.5, 1,1.5 mg/mL (labeled as groups a–f). A blank control (MEM medium only) and six replicate wells per concentration were included.

Following 24 h of treatment, cells were washed twice with PBS (pH 7.4), and 100 μL of CCK-8 reagent (10% *v*/*v* in MEM) was added to each well. After 1 h of dark incubation at 37 °C, absorbance was measured at 450 nm using a microplate reader (BioTek Synergy H1, Agilent, Santa Clara, CA, USA). Cell viability (%) was calculated as:

$$\mathrm{Cell}\;\mathrm{viability}(\%)=(\frac{A_{sample}-A_{blank}}{A_{control}-A_{blank}})\times 100$$

where *A*_control_ represents untreated cells cultured in parallel, *A*_sample_ represents test sample, *A*_blank_ represents test blank.

#### 2.5.4. Evaluation of Calcium Absorption Capacity of CCPs-SS

The calcium transport experiment was performed according to a modified protocol described by Sun [27] with the following procedures:

(1) Pre-equilibration

Fresh 6-well Transwell^®^ plates were preheated by adding 2 mL of Hanks’ Balanced Salt Solution (HBSS) to both apical and basolateral compartments, followed by incubation at 37 °C under 5% CO_2_ for 2 h.

(2) Monolayer preparation

Caco-2 cell monolayers (TEER > 400 Ω·cm^2^) were carefully removed from the incubator. The culture medium was aspirated, and monolayers were rinsed three times with pre-warmed HBSS (37 °C). Residual buffer was removed by gentle aspiration before transferring the monolayers to pre-equilibrated plates containing 2 mL of phosphate-buffered saline (PBS, pH 7.4) in each compartment. The system was stabilized at 37 °C for 30 min.

(3) Sample administration

Test compounds (calcium gluconate and calcium-chelating peptides) dissolved in transport buffer were applied to the apical chamber at a final concentration of 0.02 mg/mL.

(4) Time-dependent sampling

Basolateral aliquots (1 mL) were collected at 30-, 60-, 120-, 180-, and 240-min post-treatment. Immediately after each sampling, an equal volume of fresh PBS (37 °C) was replenished to maintain osmotic equilibrium. Calcium ion concentration in collected samples was quantified using atomic absorption spectroscopy (or specify alternative method).

### 2.6. Statistical Analysis

Analysis of variance (ANOVA) was performed using SPSS Statistics 17.0 (IBM, New York, NY, USA), and Duncan’s multiple range test was conducted at a significance level of *p* < 0.05. The graphs were drawn by OriginPro 9.0.0 (OriginLab, Northampton, MA, USA, 2019).

## 3. Results and Discussion

### 3.1. Characterization of Collagen Peptides with Varied Molecular Weights

#### 3.1.1. Changes in Amino Acid Composition of Collagen Peptides with Different Molecular Weights from the Squid Skin Hydrolysate

**Figure 1:**
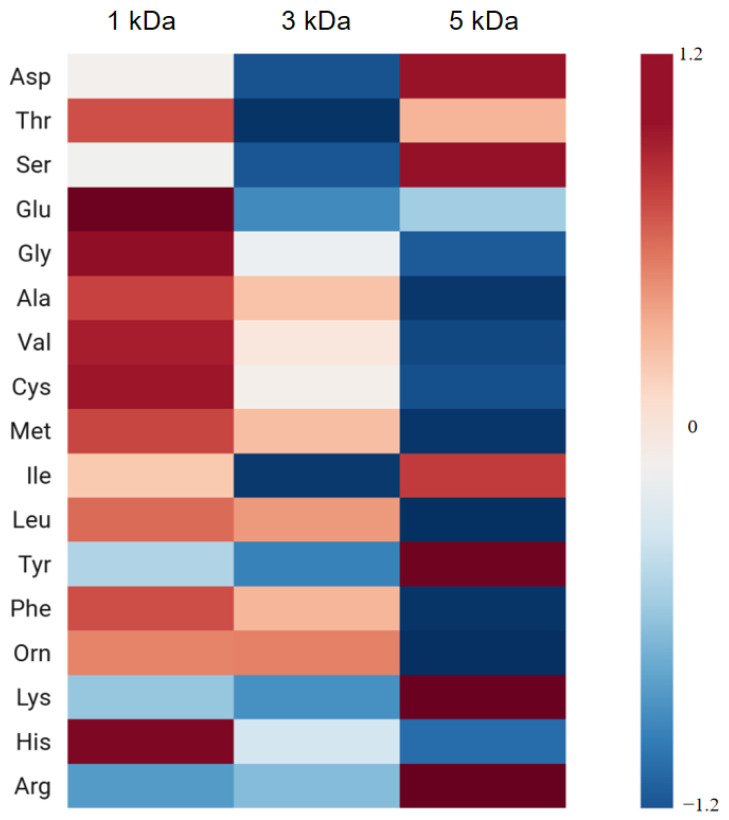
Amino acid composition of collagen peptides with different molecular weights from the squid skin hydrolysate.

After membrane separation, the proportion of amino acids with antioxidant properties in squid skin peptides with a molecular weight of less than 1 kDa was significantly higher than that in peptides treated with 3 kDa and 5 kDa membranes. Specifically, the molar concentration of phenylalanine increased from 22.44 nmol/mg to 28.15 nmol/mg, while the molar concentration of leucine increased from 36.45 nmol/mg to 57.46 nmol/mg (Figure 1). Leucine, known for its strong antioxidant capacity, not only prolongs the shelf life of food products but also promotes muscle growth.

#### 3.1.2. Hydroxyl Radical Scavenging Rate of Collagen Peptides with Different Molecular Weights from the Squid Skin Hydrolysate

**Figure 2:**
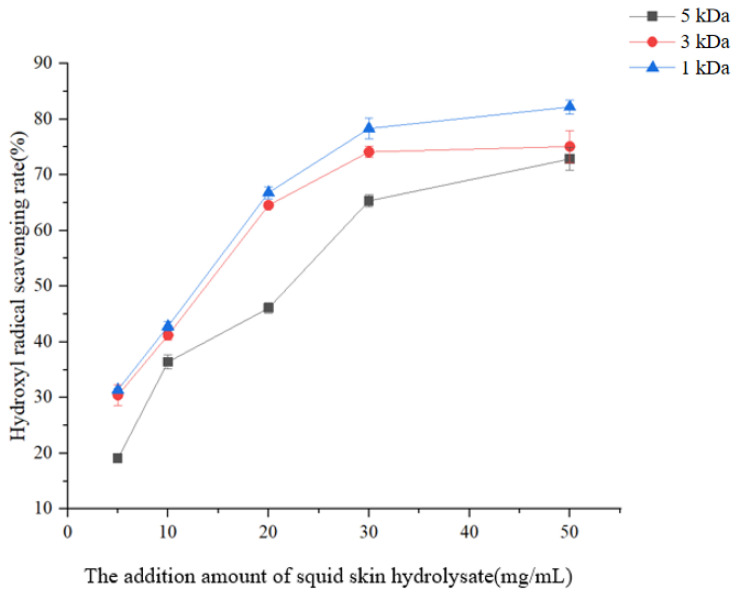
Hydroxyl radical scavenging rate of collagen peptides with different molecular weights from the squid skin hydrolysate.

The hydroxyl radical (·OH) scavenging capacity of squid skin peptides exhibited a concentration-dependent increase within the tested range (5–30 mg/mL), beyond which a plateau/reduction trend was observed (Figure 2). Notably, the <1 kDa peptide fraction demonstrated superior antioxidant performance, achieving maximal scavenging activity of 82.18 ± 1.45% at 50 mg/mL. This enhanced bioactivity correlates with the significantly higher proportion (*p* < 0.05) of antioxidant amino acids in low-MW peptides, including arginine (Arg, 12.7%), lysine (Lys, 9.2%), leucine (Leu, 8.5%), isoleucine (Ile, 6.1%), and phenylalanine (Phe, 5.8%) (Figure 1). The concentration-dependent biphasic response (activation at ≤30 mg/mL vs. decline at higher doses) may arise from peptide aggregation-induced reduced reactive site accessibility at supra-optimal concentrations or the pro-oxidant effects of metal-binding peptides under high-density conditions [28,29,30,31].

#### 3.1.3. Analysis of the Calcium Chelation Ability of Collagen Peptides with Different Molecular Weights

**Figure 3:**
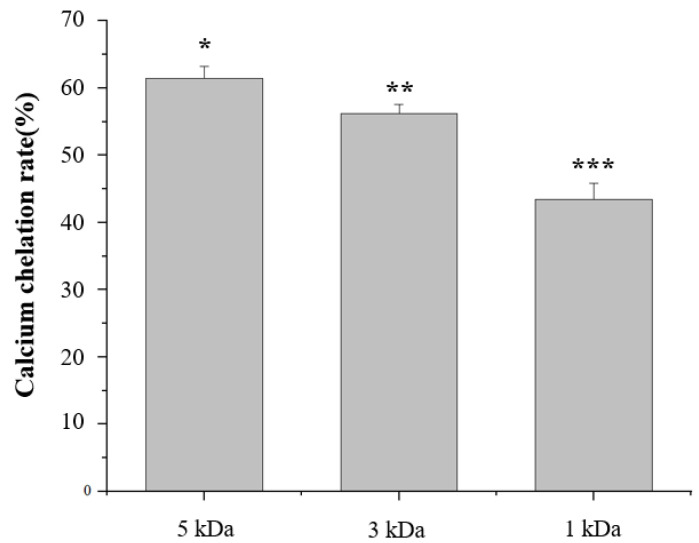
The calcium chelation ability of collagen peptides with different molecular weights. (*: *p* ≤ 0.05, **: *p* ≤ 0.01, ***: *p* ≤ 0.001).

The calcium chelation rate of squid skin peptides varied depending on the membrane separation treatment with different molecular weight cutoffs. After 1 kDa membrane separation, the calcium chelation rate reached 63.18%, which was 45.38% higher than that following 5 kDa membrane separation (Figure 3). This significant enhancement was attributed to the increased proportion of leucine. Leucine, a common chelating agent, possesses both hydrophilic and lipophilic properties, enabling it to form complexes with metal ions. Following 1 kDa membrane separation, the molar mass of leucine in the squid skin peptides reached 57.46 nmol/mg, providing additional coordination sites to bind calcium ions, thus enhancing the chelation rate between squid skin peptides and calcium ions. This result is similar to the calcium-binding peptide identified by Le [32] in the study, which contains a large number of leucine residues. In addition, leucine, as a hydrophobic amino acid, also has a strong ability to bind calcium [33]. Moreover, leucine can increase the absorption rate of calcium by influencing the opening of channels and up-regulating the expression levels of channel proteins and calcium-binding proteins, thereby achieving the effect of promoting calcium absorption [34].

### 3.2. Preparation and Optimization of the Chelating Reaction Conditions for the CCPs-SS

According to the range R, among the factors affecting the calcium content in the chelating calcium peptide of squid skin (CCPs-SS), the order of influence is chelating temperature > chelating pH > calcium chloride concentration during the chelating reaction; that is, the reaction temperature of chelating calcium peptide products is an important factor affecting the calcium content in the products (Table 3). The single-factor experiment shows that the chelating efficiency of the system begins to decrease when the temperature is higher than 60 °C. The second is the pH value of the chelation reaction and the concentration of calcium chloride during the chelation reaction. According to the results of the orthogonal test, the optimal reaction conditions for the preparation of CCPs-SS were A3, B2 and C3; namely, the concentration of calcium chloride was 12 mg/mL, the chelation reaction temperature was 60 °C, and the chelation reaction pH was 9. The results of the three-factor analysis of variance showed that the chelation temperature was significant, indicating the existence of the main effect, and the number, indicating the existence of the main effect. The specific comparison found that the primary and secondary relationship of the three factors was “temperature > pH > calcium chloride concentration”. The optimal scheme was verified and the calcium content was 65.82 mg/g, which was basically consistent with the expected result.

### 3.3. Structural Characterization of the CCPs-SS

#### 3.3.1. Zeta Potential and Particle Size of CCPs-SS

**Figure 4:**
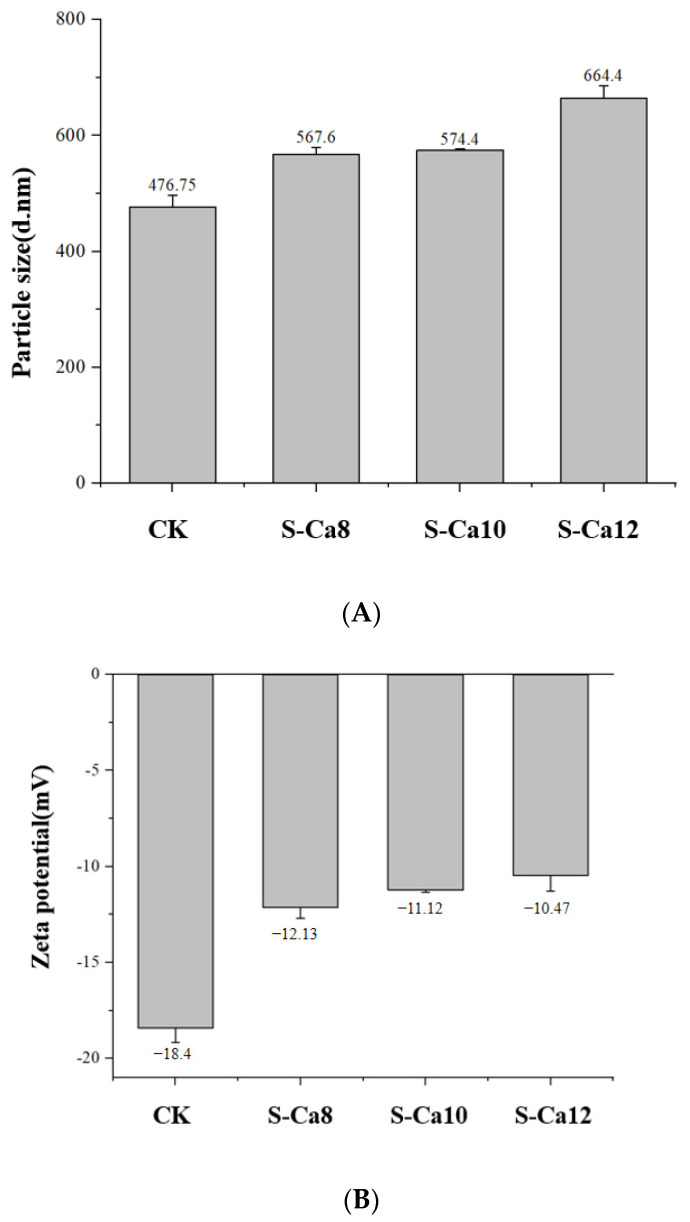
Changes in mean particle size (**A**) and zeta potential (**B**) of CCPs-SS.

After calcium chelation, the zeta potential of the squid peptide changed from −18.4 mV to −10.47 mV, indicating that Ca^2+^ was chelated with the negatively charged carboxyl group in the squid protein peptide (Figure 4A). After binding with Ca^2+^, the mean particle size of CCPs-SS increased from 476.75 nm to 664.4 nm (Figure 4B), which might be due to the fact that Ca^2+^ shielded the negative charge on the peptide chain, reduced the electrostatic repulsion between the polypeptide chains, and promoted the aggregation of peptides by forming salt bridges [35].

#### 3.3.2. Surface Morphology of CCPs-SS

**Figure 5:**
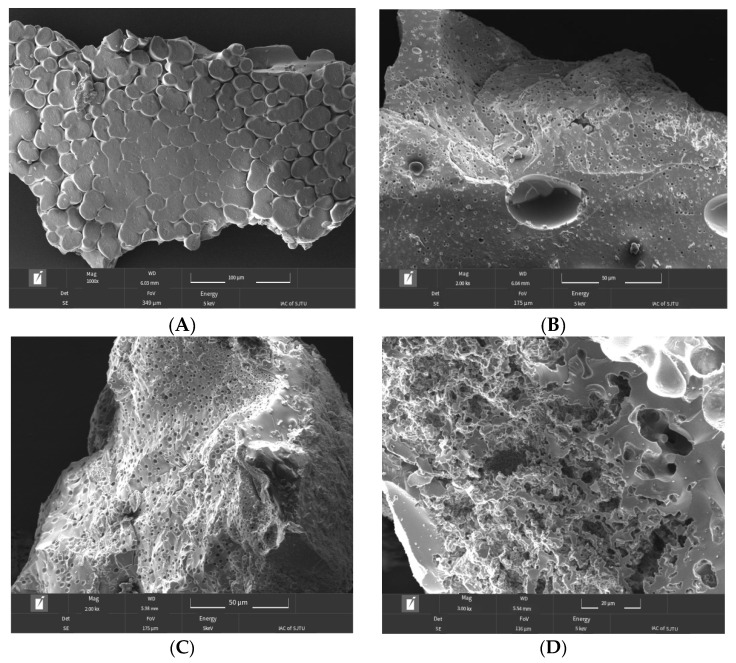
Scanning electron microscopy of squid chelating peptides at different concentrations. (**A**) CK group, (**B**) 8 mg/mL, (**C**) 10 mg/mL and (**D**) 12 mg/mL.

The squid peptide material structure is loose, the surface is smooth (Figure 5A), and after chelation with calcium ions (Figure 5B–D), the surface becomes rough. The internal structure is more compact, which is due to the coordination bond between the peptide and calcium caused by the folding of the internal structure, resulting in small granular material. These particles will attract each other and then produce aggregation. In addition, there are some cracks on the surface of the chelate. It may be that the sample absorbs moisture in the air when spraying platinum before the sample measurement. This difference may be due to the folding and aggregation of the internal structure caused by the coordination bond formed by the binding of the squid skin peptide to Ca^2+^ [36], which destroyed the original dense structure of the squid skin peptide. In addition, the bridging effect formed by the combination of amino and carboxyl groups in peptides with calcium can also change the surface [37]. Wang et al. also observed similar morphological characteristics in the microscopic morphology of peptide-chelated calcium in cucumber seeds [38]. Hou et al. also reported in the study of desalted duck egg peptide-chelated calcium that the presence of calcium ions would lead to peptide folding and aggregation, leading to the formation of peptide calcium chelates [38] and the environment of the electron microscope is a vacuum, which leads to the rapid loss of water and dryness of the sample, leaving traces.

#### 3.3.3. The Infrared Spectroscopy of CCPs-SS

When a peptide is irradiated with infrared light at continuously varying frequencies, molecules absorb infrared light at specific frequencies, transitioning from the ground state to an excited state. This absorption weakens the transmitted light in these regions [39]. Polypeptides contain various functional groups, such as carboxyl, carbonyl, and amino groups, which can coordinate with metal ions. By comparing the shifts in characteristic group absorption peaks between the native polypeptide and the peptide-calcium chelate, the binding site of the peptide-calcium complex can be inferred.

**Figure 6:**
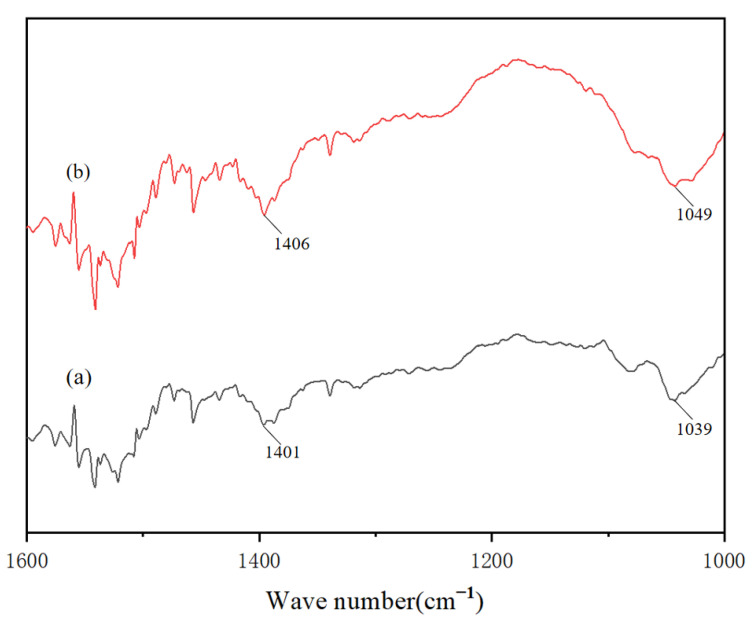
Infrared spectrum changes before (**a**) and after (**b**) chelation.

Infrared spectral analysis revealed significant shifts in characteristic absorption peaks following calcium chelation (Figure 6). Notably, the hydroxyl (-OH) stretching vibration shifted from 1039 cm^−1^ in native squid peptides to a broadened peak at 1049 cm^−1^ post-chelation, indicating altered hydrogen bonding patterns. Additionally, the carboxyl (-COOH) asymmetric stretching vibration exhibited a 5 cm^−1^ bathochromic shift from 1401 cm^−1^ to 1406 cm^−1^, suggesting deprotonation and covalent coordination between the carboxylate (-COO^−^) and Ca^2+^ ions to form a stable -COO-Ca^+^ structure. This spectral evolution is consistent with the ion exchange mechanism, where Ca^2+^ displaces H^+^ at carboxyl sites, aligning with previous reports on metal–peptide chelation thermodynamics [40].

### 3.4. Cell Toxicological Assay (CCK-8) for CCPs-SS

**Figure 7:**
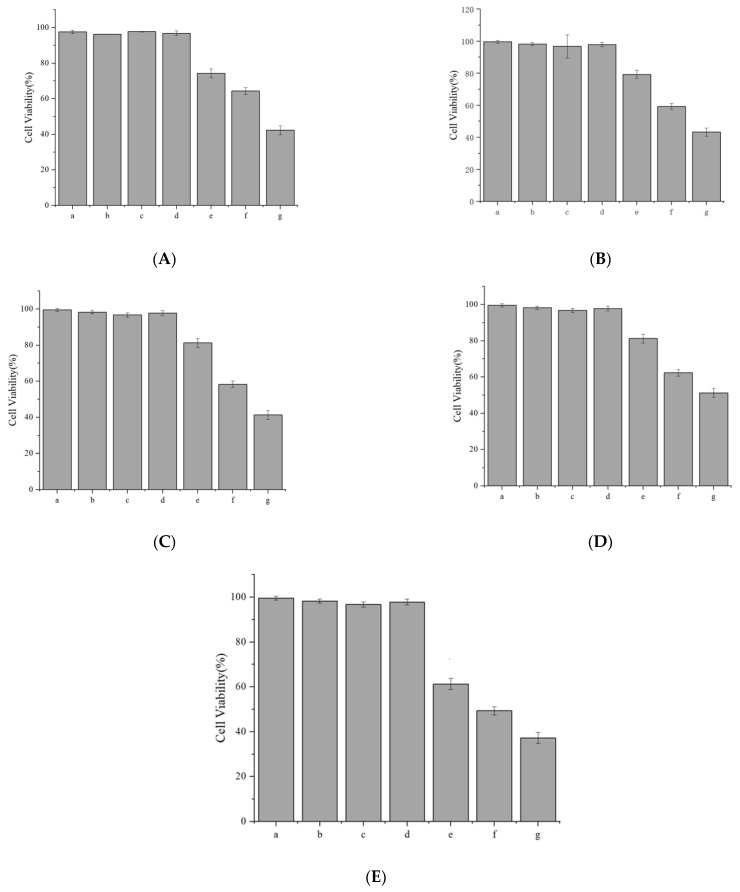
Cell viability at different administered concentrations, where: (**A**) CaCl_2_, (**B**) Calcium gluconate, (**C**) CCPs-SS (1 K), (**D**) CCPs-SS (3 K), (**E**) CCPs-SS (5 K).

After the addition of calcium chelate for 24 h, the cell survival rate of each group was shown in Figure 7. It was found that the cell survival rate began to decline when the concentration was above 0.2 mg/mL, and the larger concentration affected the growth of cells. This may be because when the cells were exposed to a high calcium environment, the mitochondria began to absorb too much calcium, resulting in the stop of ATP synthesis and depolarization of the mitochondrial inner membrane. This results in a decline in cell survival.

### 3.5. Evaluation of the Calcium Absorption Capacity of Squid Skin Peptides

**Figure 8:**
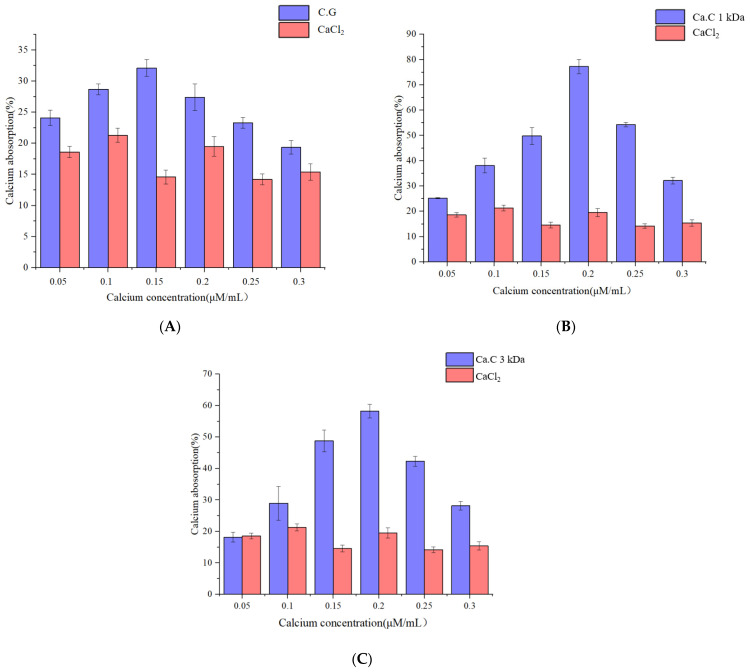
Calcium absorption ability of squid skin chelating peptides with different molecular weights, where (**A**) is (a) inorganic calcium control group; (**B**) for squid skin peptides with molecular weight less than 1 K; (**C**) is squid skin peptide with molecular weight less than 3 K.

The retention of calcium in a soluble form within the intestinal lumen is essential for its absorption, typically as free calcium ions or in the chelate form of soluble organic molecules. However, dietary calcium often fails to meet the physiological needs of the human body due to insufficient intake and low bioavailability. Chelating calcium ions with peptides can enhance calcium bioavailability. Studies (Figure 8A–C) have shown that chelated calcium peptides from squid skin, with a molecular weight below 1 kDa, exhibit the highest calcium absorption capacity, reaching 77.14%. Nevertheless, the calcium absorption capacity of cells decreases as the molecular weight of the squid peptides increases. Overall, the calcium absorption capacity of calcium-binding peptides after chelation surpasses that of inorganic calcium. This result is consistent with Chen’s [41] research results, where the blood calcium concentration of mice fed with chelated bonito protein peptide-chelated calcium was higher than that of mice fed with calcium carbonate.

## 4. Conclusions

Squid skin was used as the raw material and subjected to enzymatic hydrolysis using alkaline protease, followed by processing through a membrane separation device. This resulted in the separation of three types of squid skin peptides with varying molecular weights. After calcium ion chelation, squid skin calcium peptide chelates were obtained, and a preparation process for these chelates was established. Scanning electron microscopy (SEM), amino acid analysis, and particle size analysis revealed that the squid skin chelating peptides (CCPs-SS) with a molecular weight below 1 kDa exhibited the best antioxidant properties and calcium chelating rates. This superior performance may be attributed to the relatively high proportion of leucine in these peptides. As a hydrophobic amino acid, leucine is active in chelation reactions. When the molar concentration of leucine increased from 36.45 nmol/mg to 57.46 nmol/mg, the chelation rate of CCPs-SS increases by 45.38% to 63.18%. Additionally, an evaluation model for cellular calcium absorption capacity demonstrated that the squid skin chelating calcium peptides with a molecular weight below 1 kDa had the highest calcium absorption capacity, achieving 77.14%. This study enhances the bioavailability of calcium and may achieve certain feasibility in the application of natural calcium supplement products in the future. It provides a certain working basis for enhancing product utilization and expanding the development of new calcium supplement products.

## Figures and Tables

**Table 1:** Orthogonal experimental design of reaction conditions for preparation of the CCPs-SS.

Levels	pH	Chelation Temperature (°C)	Calcium Chloride Concentration (mg/mL)
1	6	55	8
2	7	60	10
3	8	65	12

**Table 2:** Orthogonal test design.

	A	B	C
Experimental Group	pH	Chelation Temperature (°C)	Calcium Chloride Concentration
1	1 (6)	1 (55 °C)	1 (8 mg/mL)
2	1	2	2
3	1	3	3
4	2 (7)	1 (60 °C)	2 (10 mg/mL)
5	2	2	3
6	2	3	1
7	3 (8)	1 (65 °C)	3 (12 mg/mL)
8	3	2	1
9	3	3	2

**Table 3:** Results of the orthogonal test for optimizing the chelation conditions of the CPs-SS.

Column	A	B	C	
Factors	CaCl_2_ Concentration (mg/mL)	Chelation Temperature (°C)	Chelation pH	Calcium Content (mg/g)
Experiment 1	1 (8)	1 (50 ° C)	1 (7)	36.79 ± 0.62
Experiment 2	1	2 (60)	2 (8)	64.72 ± 1.02
Experiment 3	1	3 (70)	3 (9)	14.93 ± 0.26
Experiment 4	2 (10)	1	2	28.39 ± 0.17
Experiment 5	2	2	3	34.21 ± 0.81
Experiment 6	2	3	1	22.22 ± 0.14
Experiment 7	3 (12)	1	3	41.56 ± 0.32
Experiment 8	3	2	1	24.73 ± 0.68
Experiment 9	3	3	2	32.92 ± 0.44
K_1_	116.45	106.75	83.75	
K_2_	84.82	123.66	126.03	
K_3_	99.21	70.07	90.7	
R	31.63	53.59	42.28	

## Data Availability

The original contributions presented in the study are included in the article, further inquiries can be directed to the corresponding author.
